# A Delphi study and ranking exercise to support commissioning services: future delivery of Thrombectomy services in England

**DOI:** 10.1186/s12913-018-2922-3

**Published:** 2018-02-22

**Authors:** Kristoffer Halvorsrud, Darren Flynn, Gary A. Ford, Peter McMeekin, Ajay Bhalla, Joyce Balami, Dawn Craig, Phil White

**Affiliations:** 1 Institute of Health and Society Newcastle University, Newcastle Upon Tyne, UK; 2 Institute of Neuroscience, Newcastle University, 3-4, Claremont Terrace, Newcastle upon Tyne, NE2 4AX UK; 3 School of Health, Community and Education Studies, Northumbria University, Newcastle Upon Tyne, UK; 4 Guy’s and St Thomas’ NHS Foundation Trust, London, UK; 5 Newcastle upon Tyne Hospitals NHS Foundation Trust, Newcastle Upon Tyne, UK; 6 Centre for Evidence Based Medicine, University of Oxford, Oxford, UK; 7 Oxford University Hospitals NHS Trust and Oxford University, Oxford, UK; 8 Barts and The London School of Medicine and Dentistry, Queen Mary University of London, London, UK

**Keywords:** Delphi exercise, Service organisation, Consensus, Neurointervention, Intra-arterial thrombectomy

## Abstract

**Background:**

Intra-arterial thrombectomy is the gold standard treatment for large artery occlusive stroke. However, the evidence of its benefits is almost entirely based on trials delivered by experienced neurointerventionists working in established teams in neuroscience centres. Those responsible for the design and prospective reconfiguration of services need access to a comprehensive and complementary array of information on which to base their decisions. This will help to ensure the demonstrated effects from trials may be realised in practice and account for regional/local variations in resources and skill-sets. One approach to elucidate the implementation preferences and considerations of key experts is a Delphi survey. In order to support commissioning decisions, we aimed using an electronic Delphi survey to establish consensus on the options for future organisation of thrombectomy services among physicians with clinical experience in managing large artery occlusive stroke.

**Methods:**

A Delphi survey was developed with 12 options for future organisation of thrombectomy services in England. A purposive sampling strategy established an expert panel of stroke physicians from the British Association of Stroke Physicians (BASP) Clinical Standards and/or Executive Membership that deliver 24/7 intravenous thrombolysis. Options with aggregate scores falling within the lowest quartile were removed from the subsequent Delphi round. Options reaching consensus following the two Delphi rounds were then ranked in a final exercise by both the wider BASP membership and the British Society of Neuroradiologists (BSNR).

**Results:**

Eleven stroke physicians from BASP completed the initial two Delphi rounds. Three options achieved consensus, with subsequently wider BASP (97%, *n* = 43) and BSNR members (86%, *n* = 21) assigning the highest approval rankings in the final exercise for transferring large artery occlusive stroke patients to nearest neuroscience centre for thrombectomy based on local CT/CT Angiography.

**Conclusions:**

The initial Delphi rounds ensured optimal reduction of options by an expert panel of stroke physicians, while subsequent ranking exercises allowed remaining options to be ranked by a wider group of experts within stroke to reach consensus. The preferred implementation option for thrombectomy is investigating suspected acute stroke patients by CT/CT Angiography and secondary transfer of large artery occlusive stroke patients to the nearest neuroscience (thrombectomy) centre.

**Electronic supplementary material:**

The online version of this article (10.1186/s12913-018-2922-3) contains supplementary material, which is available to authorized users.

## Background

There is an accruing body of evidence from multiple well conducted randomised controlled trials (RCTs) illustrating the benefits of intra-arterial mechanical thrombectomy (IAT) compared with intravenous thrombolysis in acute ischaemic stroke caused by large artery occlusion. Recanalisation occurs in ~ 30% (range 10 to > 45%) of large artery occlusive strokes (LAOS) after treatment with intravenous thrombolysis [1, 2], whereas IAT with contemporary devices achieves good recanalisation in > 70% in patients with LAOS [3]. Level 1 evidence from multiple RCTs, including one in the UK [4–11], demonstrates that IAT in patients with anterior circulation acute LAOS is safe and efficacious within six hours of stroke onset. Meta-analyses have confirmed an increased likelihood of improved functional outcomes (mRS 0–2) at 90 days associated with IAT, with no effect on mortality or symptomatic intracranial haemorrhage [12–15]. Trial data are however almost entirely based on vascular imaging triage (Computed Tomographic or Magnetic Resonance angiography of the brain) leading to IAT delivered by experienced neurointerventionists working in established teams in neuroscience centres [5, 6, 8]. There is no level 1 data on outcomes of thrombectomy performed by non neurointerventionists.

Currently very limited numbers of centres can deliver IAT substantial variability in IAT service provision in England as found in a survey of current interventional neuroradiology (INR) services in England (2014 population ~ 54 million) [16]. The financial and workforce constraints resulting in the variable provision and performance of INR (and other clinical) services in England, have instigated calls for service reconfiguration and the identification of cost-effective models for optimal provision across the country. There has been considerable debate over proposed or implemented service reconfigurations within the National Health Service (NHS) in England [17]. These types of decisions are not fully informed by trial evidence and there is often a lack of agreement between stakeholders, with limited options available to make the decision more objective. To help inform decision making about IAT service provision, a further appreciation of the preferences of clinical experts is required. These preferences should form part of the information underpinning the evidence base, which should be clearly presented to decision makers responsible for the design and implementation of service delivery policies within stroke care.

In order to support commissioning decisions, we aimed to use an electronic Delphi survey to establish consensus on the options for the future organisation of thrombectomy services in England amongst physicians with clinical experience in managing large artery occlusive stroke; in the process helping demonstrate the feasibility of the methods for informing service delivery policy in other areas. We designed a Delphi and ranking exercise to to focus on questions of “what could/should be” relating to the organisation of health care services [18–22]. The purpose of the study was to derive consensus on thrombectomy service organisation. The Delphi technique makes use of multiple iterations of responses to questionnaires from a panel of experts to establish consensus on a particular topic; multiple iterations (rounds) provide the panellists with opportunities to reflect on feedback on their responses to statements in previous iterations [18]. The Delphi survey was delivered to physicians from NHS stroke clinical services that are practising 24/7 intravenous (IV) thrombolysis. Data generated from this process will later be used to inform the development of a health economic model to estimate the effectiveness and cost-effectiveness of different models of IAT services for the treatment of LAOS.

## Methods

The data collection period for our study was November 2015 to January 2017. Participation was anonymous. Contacts at the respective professional bodies were aware of the identity of responders, but not the research team. Contacts at the respective professional bodies forwarded anonymised completed forms to the research team via email.

### Procedure for Delphi exercise

An request was sent to the chair of the British Association of Stroke Physicians (BASP) Clinical Standards Committee to disseminate a request for participation to members. Stroke physicians currently employed within an NHS stroke clinical service that delivers 24/7 IV thrombolysis and were part of the BASP Clinical Standards and/or Executive Membership were eligible to participate in the initial stage. The initial recruitment of these stroke physicians for the Delphi exercise comprised a purposive sampling strategy predicated on the grounds that – as part of their specialist training and direct involvement in stroke care on a daily basis – they possess critical knowledge and multidisciplinary team skills in the diagnosis and treatment of stroke. Based on the literature [26, 27], we aimed to recruit between 15 and 25 panellists who could provide a representative pooling of ratings assigned to the options for future organisation of thrombectomy services. The initial Delphi exercise served to delineate the options subsequently presented in a ranking exercise (see later), which engaged a wider range of relevant experts managing LAOS and to strengthen the validity of our results by converging towards consensus on one proposition.

With reference to relevant literature [1–15], policy/guidelines [23–25] and discussions within the study team, a survey questionnaire was initially developed with 12 options (the propositions) for future organisation of thrombectomy services in England (see Table 1).

**Table 1:** Options (propositions) for Thrombectomy Service Provision

Below we present a description of 12 potential options for delivering thrombectomy with an explanatory footnotes (where required). Please score each of the 12 options using a 7-point Likert scale:
1. Any local provider “ad hoc”^a^
2. Any local provider delivers IAT on a formal rota^b^
3. Transfer to nearest primary coronary percutaneous intervention unit and cardiology manage^c^
4. Transfer to nearest primary coronary percutaneous intervention unit and shared care with stroke physicians^d^
5. Ambulance bypass for all acute stroke patients of known time onset to comprehensive stroke unit where advanced imaging and “expert intra-arterial thrombectomy [IAT]” are available 24/7^e^
6. Local CT and transfer all patients with NIHSS ≥10 to the nearest neuroscience centre for interventional neuroradiologist delivered “expert thrombectomy”^f^
7. Local CT/CTA then transfer all large artery occlusive stroke patients to nearest neuroscience centre for interventional neuroradiologist delivered “expert thrombectomy”^g^
8. Local advanced imaging then selective transfer to nearest neuroscience centre for “expert thrombectomy”^h^
9. Local CT/CTA then transfer large artery occlusive stroke patients to nearest neuroscience centre for advanced imaging and “expert thrombectomy”
10. Advanced imaging performed locally but interpreted centrally by Neuroradiology then selective transfer to nearest neuroscience centre for “expert thrombectomy”
11. Selective transfer to nearest on call neuroscience centre for “expert thrombectomy”^i^
12. Interventional neuroradiologist and necessary support team on standby in Neuroscience centre – they transfer to patient’s hospital to deliver expert intra-arterial thrombectomy when large arterial occlusion stroke is confirmed^j^

^a^Any physician with some intra-arterial catheter skills delivers intra-arterial thrombectomy [IAT] as best they can when they can. There is no level I evidence (obtained from at least one properly designed & conducted randomised controlled trial) for this option

^b^Interventional radiologists would likely be at the core of this option. There is no level I evidence for this option

^c^There is no level I evidence for this option

^d^Where a primary coronary percutaneous intervention unit and an acute stroke unit are geographically close enough to allow this to be feasible

^e^According to data from the Sentinel Stroke National Audit Programme (SSNAP) 70% of acute stroke patients have known time onset and 60% of those reach hospital within 4 h = 42%; 12% in SSNAP are haemorrhage not ischaemic strokes

^f^This option is sometimes called a “drip and ship” approach; The neuroscience centre team might include interventional neuroradiology trained/mentored interventional radiologists or cardiologists to facilitate a 24/7 service

^g^37% of all stroke patients arrive at hospital within 4 h with ischaemic stroke of known onset time. ~ 50% of patients have large artery occlusive strokes. So IAT currently potentially applies to almost 20% of acute disabling ischaemic strokes; Adjunctive IAT approach is proven (level 1 evidence) to increase mRS 0–2 by 12% to 14% with benefit across the Rankin scale of shift to reduced disability

^h^Selective brain tissue viability assessment approach to IAT is proven (level 1 evidence) to increase mRS 0–2 by 24% to 31% with benefit across the Rankin scale of shift to reduced disability; All RCT results are based on expert interpretation of advanced imaging as triage for intra-arterial thrombectomy; This option is a less time critical approach

^i^This entails networking of Interventional Neuroradiology units to deliver 24/7 cover sooner – with some longer transfer times, but does mean the efficacy data from RCTs can be applied (underpinned by data for UK centres from the PISTE trial)

^j^This is provided by very few places worldwide; This model of provision is clearly very expensive

Piloting of the questionnaire was undertaken with members of the research team and collaborating stroke physicians. Participation was anonymous and correspondence was managed by email using panellists’ BASP reference numbers through the BASP secretariat. No more than three rounds were planned, in order to ensure the process did not become too repetitive and time-consuming to maintain an adequate response rate [18]. After the second Delphi round, three options fulfilled our pre-set criteria for establishing consensus and were rated in the final ranking exercise (see below).

In the first Delphi round each panellist was asked to assign ratings using a 7-point Likert scale (1 = very strongly disapprove to 7 = very strongly approve) to each of the 12 options. Panellists were also given the opportunity to provide free text comments on the 12 options at the end of the questionnaire.

In the subsequent Delphi round, panellists were informed of aggregate-level summary statistics (mode, median and interquartile range) for the 12 options, including (i) the individual ratings they had assigned to each option in the previous round; and (ii) a brief summary of any free text comments. In addition, propositions with aggregate scores falling within the lowest quartile from the first Delphi round (including all values of 1, 2 and 3 on the Likert scale that represent very strongly disapprove, quite strongly disapprove and disapprove, respectively) were removed from subsequent rounds. Panellists in the second Delphi round were asked to consider their ratings for the remaining propositions with reference to the data from the previous round.

Consensus for “approval” was defined as ≥75% of the panellists’ ratings for each option falling within three categories (approve, quite or very strongly approve) on the 7-point Likert scale. A period of two weeks was allocated for panellists to respond to each round. Our cut-off points for removal of propositions from subsequent rounds and consensus were consistent with other Delphi studies [26, 27, 29, 30].

### Procedure for ranking exercise

Following the two-round Delphi exercise with BASP Clinical Standards and/or Executive members from NHS stroke clinical services that deliver 24/7 IV thrombolysis, propositions that reached consensus for approval were subjected to a ranking exercise to establish wider professional stroke physicians’ (see Additional file 1: Appendix 1) and neuroradiologists’ (see Additional file 2: Appendix 2) preferences on the remaining options.

A request for participation was sought from any member of the BASP currently employed within the English NHS with clinical experience in managing LAOS. The BASP encompasses members from a wide range of clinical backgrounds (e.g. stroke, general medicine, geriatric, neurology), with the association itself valuing this diversity and the range of roles and services within stroke care that its members provide [28]. As such, opening the ranking exercise up to *any* member of the BASP highlights an inclusive approach (and validates a degree of generalisability of opinions) of those who deliver stroke care in England.

In addition, a parallel ranking exercise was conducted with full members of the relevant special interest group of the Royal College of Radiologists, the British Society of Neuroradiologists (BSNR), currently employed within an NHS service in the UK that delivers stroke imaging. The view was taken that clinical neuroradiologists – due to the remaining options all including the use of various brain imaging techniques – would be particularly well positioned, alongside the BASP membership, to help reach consensus on options for the future delivery of thrombectomy services.

Information on the outcomes of the preluding Delphi exercise was provided to the wider BASP and BSNR members participating in the ranking exercise. Participation was anonymous and correspondence with panellists was managed by the Secretariat of professional societies’ using reference numbers.

The wider BASP and BSNR membership were asked to rank options using a 7-point Likert scale (1 = very strongly disapprove to 7 = very strongly approve). Respondents were asked, in ranking, to use their experience and judgement to consider the availability, practicality/deliverability and potential cost of each of the different options. A free text box to write down any comments was provided in both surveys. Two weeks were given to members of the two professionals groups to respond before a reminder was sent out with an additional week before the survey was closed.

Summary statistics (frequencies, percentage frequencies, median and IQR) for rankings assigned to options by respondents from the wider BASP and BSNR were calculated (see Tables 2 and 3 and Additional file 3: Appendix 3). Free text responses associated with options assigned high approval ratings were subjected to a conceptual content analysis (see Additional file 4: Appendix 4). This involved collating free text comments made for options with high approval ratings. Emergent coding was then used to summarise key themes for discussion between two authors (DF and KH) who agreed on final themes, which were peer-reviewed by the wider research team. Final themes were supported by quotations, as well as to permit readers to ascertain for themselves their validity.

**Table 2:** Aggregate panellist responses (Likert Scale category) for each proposition, *N* = 11

	Percentage Responses						
Proposition Number (from original list in Table 1)	1 very strongly disapprove	2 quite strongly disapprove	3 disapprove	4 neutral	5 Approve	6 quite strongly approve	7 very strongly approve
2. Any local provider delivers IAT on a formal rota		**55**	**18**		**27**		
4. Transfer to nearest primary coronary percutaneous intervention unit and shared care with stroke physicians	**18**	**18**	**37**	**18**			**9**
5. Ambulance bypass for all acute stroke patients of known time onset to comprehensive stroke unit where advanced imaging and “expert intra-arterial thrombectomy [IAT]” are available 24/7		**9**	**55**	**18**	**18**		
6. Local CT and transfer all patients with NIHSS ≥10 to the nearest neuroscience centre for interventional neuroradiologist delivered “expert thrombectomy” **			**9**	**27**	**27**	**27**	
**7.** Local CT/CTA then transfer all large artery occlusive stroke patients to nearest neuroscience centre for interventional neuroradiologist delivered “expert thrombectomy” **					**27**	**37**	**27**
8. Local advanced imaging then selective transfer to nearest neuroscience centre for “expert thrombectomy”			**18**	**18**	**18**	**37**	**9**
9. Local CT/CTA then transfer large artery occlusive stroke patients to nearest neuroscience centre for advanced imaging and “expert thrombectomy”				**18**	**18**	**46**	**18**
10. Advanced imaging performed locally but interpreted centrally by Neuroradiology then selective transfer to nearest neuroscience centre for “expert thrombectomy”			**9**	**27**	**9**	**46**	**9**
11. Selective transfer to nearest on call neuroscience centre for “expert thrombectomy”					**36**	**46**	**18**

**N = 10

*NB* Propositions that achieved consensus approval have been highlighted in bold text. Percentages may not equal 100 due to rounding

**Table 3:** Summary of results from ranking exercises

	*Wider BASP members (N = 43)* Percentage Responses						
Using your experience and judgement, please take the following elements into consideration when assigning scores to the options: availability; practicality/deliverability; and cost (including of any additional software or hardware likely to be required in your region)	1 very strongly disapprove	2 quite strongly disapprove	3 disapprove	4 neutral	5 approve	6 quite strongly approve	7 very strongly approve
1. Patients with large artery occlusive stroke are transferred to nearest [neuroscience] centre for thrombectomy based on local CT/CTA alone^a^		**2**			**21**	**53**	**23**
2. Patients are transferred to nearest [neuroscience] centre for thrombectomy based on advanced imaging obtained at referring hospital^b^	**12**	**5**	**16**	**23**	**21**	**16**	**7**
3. Selective transfer to nearest on call [neuroscience] thrombectomy centre for expert thrombectomy^c^	**16**	**19**	**12**	**19**	**14**	**12**	**9**
Using your experience and judgement, please take the following elements into consideration when assigning scores to the options: availability; practicality/deliverability; and cost (including of any additional software or hardware likely to be required in your region) Whilst options 2 & 3 are both “Advanced Imaging Triage” they may differ in deliverability, cost & practicality so they have been separated out for this exercise. There is of course uncertainty over the strength of evidence supporting either option	*Full members of the BSNR (N = 21)* Percentage Responses						
1. Patients are transferred for thrombectomy based on local CT/CTA alone^d^	**5**		**5**	**5**	**19**	**29**	**38**
2. Patients are transferred for thrombectomy based on formal ASPECTS & Collateral Scoring in addition to confirming large artery occlusion present - “Advanced Imaging Triage ACS”e**			**24**	**29**	**14**	**14**	**14**
3. Patients are transferred for thrombectomy based on CT Perfusion parameters in addition to confirming large artery occlusion present - “Advanced Imaging Triage PERFUSION”^f^**	**14**	**19**	**38**	**14**		**10**	
4. Selective transfer to nearest on call neuroscience centre for “expert thrombectomy”^g^		**5**	**29**	**5**	**29**	**5**	**29**

**N = 20

^a^ *37% of all stroke patients arrive at hospital within 4 h with ischaemic stroke of known onset time. ~ 40–50% of patients have large artery occlusive strokes*

*Adjunctive IAT approach is proven (level 1 evidence) to increase mRS 0–2 by 12% to 14% with benefit across the Rankin scale of shift to reduced disability*

Facilities will need to be available for the neurointerventionist to rapidly review CT/CTA prior to accepting a referral. This may require additional IT infrastructure

Responsibility for formal reporting will be with the centre acquiring the CT/CTA images unless other contractual arrangements are formally agreed

^b^ *Selective brain tissue viability assessment approach to IAT is proven (level 1 evidence) to increase mRS 0–2 by 24% to 31% with benefit across the Rankin scale of shift to reduced disability. All RCT results are based on expert interpretation of advanced imaging as triage for intra-arterial thrombectomy. Facilities will need to be available for the neurointerventionist to rapidly review imaging prior to accepting a referral. This will require additional IT infrastructure. Responsibility for formal reporting will be with the centre acquiring the imaging unless other contractual arrangements are formally agreed*

^c^This is a flexible clinical judgement driven referral route – so that for example if plain CT shows an obvious hyper-dense MCA sign, the ASPECTS score is good (7+) & NIHSS is ≥6, referral for thrombectomy is made without CTA if obtaining such locally would add significant delays. However, this may add delay downstream in the pathway for thrombectomy as a second CT scanner visit will be required on arrival at receiving hospital. *This may entail networking of Neurorinterventional units to deliver 24/7 cover sooner- with some longer transfer times, but does mean the efficacy data from RCTs can be applied (underpinned by data for UK centres from the PISTE trial)*

^d^Facilities will need to be available for the neurointerventionist to rapidly review these prior to accepting a referral. This may require additional IT infrastructure. Responsibility for formal reporting will be with the centre acquiring the CT/CTA images unless other contractual arrangements are formally agreed

^e^This reflects evidence of ESCAPE trial. Footnotes above also apply to all these options

^f^This reflects evidence of EXTEND/SWIFT PRIME trials. Footnotes to option 1 also apply. This may require a region wide adoption of a standardised protocol & software such as RAPID or OLEA

^g^This is a flexible clinical judgement driven referral route – so that for example if plain CT shows an obvious hyper-dense MCA sign, the ASPECTS score is good (7+) & NIHSS is ≥6, referral for thrombectomy is made without CTA, which may add delay to the pathway to thrombectomy

*NB* The propositions that reached consensus from the respective groups have been highlighted in bold text. Percentages may not equal 100 due to rounding

## Results

### Delphi exercise

Fifteen panellists from BASP completed the first Delphi round. They comprised an experienced sample - with 67% having practiced as a stroke consultant for more than 10 years. Panellists worked across all regions of England (see Additional file 5: Appendix 5).

After the first Delphi round, the following three propositions had aggregate scores within the lowest quartile indicating “disapproval” (see Table 1 for the propositions): *Proposition 1* (87% very/quite strongly disapprove or disapprove); *Proposition 3* (87% very/quite strongly disapprove or disapprove); *Proposition 12* (60% very/quite strongly disapprove or disapprove).

Eleven of the 15 panellists from the first round also completed the second round of the Delphi exercise and rated the remaining nine propositions (Table 2).

Following the second Delphi round, consensus “approval” was achieved for three propositions (see Table 1 for the propositions): *Proposition 7* (91% very/quite strongly approve or approve); *Proposition 9* (82% very/quite strongly approve or approve); *Proposition 11* (100% very/quite strongly approve or approve).

### Ranking exercises with wider BASP membership and BSNR

The wider BASP members were asked to rank the three options that reached consensus approval in the two rounds of the Delphi exercise. Propositions 7 and 9 were designated as *simple imaging* and *advanced imaging* driven options, respectively, with proposition 11 designated as a *clinical judgement driven* option in the final ranking exercise.

BSNR members were also asked to rank these options. However, due to their routine imaging practices in their role as neuroradiologists, the advanced imaging driven option was dichotomised into two options relating to technical imaging pathways driven by (i) the Alberta Stroke Program Early CT Score (ASPECTS) and collateral scoring or (ii) computed tomography (CT) perfusion parameters.

The wider BASP membership returned 43 surveys (representing approximately 15% of the total membership). The respondents had a range of experience - number with 0–5 years, 5–10 and 10+ years of experience as a stroke physician was 10 (23%), 11 (26%) and 22 (51%), respectively. The majority (*n* = 34, 79%) currently had arrangements in place to refer patients for thrombectomy (*n* = 14, 41% had formal arrangements and *n* = 20, 59% had ad hoc arrangements). The BASP respondents came from all regions of England.

A total of 21 responses were received from BSNR members (also representing approximately 15% of all BSNR members in the UK) with 62% practicing 10+ years and 71% of respondents’ currently undertaking regular cerebral vascular interventions. All regions of the UK were represented.

Responses from the wider BASP and the BSNR members are summarised in Table 3. There was a clear consensus (97% of wider BASP and 86% of BSNR members very/quite strongly approved or approved) for a simple imaging driven pathway: *patients with large artery occlusive stroke are transferred to nearest [neuroscience] centre for thrombectomy based on local CT/CT Angiography alone*.

## Discussion

Currently in England all thrombectomy is provided by neurointerventionists - almost exclusively based in Regional Neuroscience Centres. This Delphi and ranking exercise by groups of practising NHS clinicians with regular clinical expertise in the management of LAOS patients has considered implementation options for thrombectomy into routine stroke care pathways in England. The preferred option is conveying suspected stroke patients to the nearest hospital with a hyperacute acute stroke unit for CT/CT Angiography brain imaging investigation and secondary transfer of those identified as LAOS to nearest [neuroscience] thrombectomy centre. Whilst the discussion focuses on the LAOS scenario presented, the methods used have the potential to be utilised when considering the implementation of any new service/treatment.

### Strengths and limitations

This study provides policymakers and practitioners with complementary data that previous trials on the effectiveness of thrombectomy services have failed to capture, employing a proven methodology in use since the 1950s [29, 30]. The initial Delphi rounds ensured the optimal reduction of options by an expert panel of stroke physicians, while subsequent ranking exercises allowed remaining options to be ranked by a wider group of experts. The iterative procedure with multiple rounds contributed to enhancing the accuracy of results by converging towards consensus in favour of one option.

The results of *any* Delphi and ranking exercise can be susceptible to the interpretations and specific proposition formulations of the study team which – if somewhat differently worded – might have nuanced the findings [29]. This issue is accentuated by the fact that participants are asked to rank pre-formulated and closed options. However, we believe a strength of this study is that a wide range of options (*n* = 12) were specified from its inception. These options were informed by existing research and policy guidelines on thrombectomy, in addition to the attention to detail and extensive discussions within an experienced research team. Although recognising that some of these options are not equally supported by available evidence and/or may be less feasible in clinical practice, we included all of them as the purpose of this Delphi and ranking exercise was to eliminate less effective, less deliverable and/or less cost-effective options by consensus iteration from a wider group of experts, rather than biasing the process by including fewer options favoured by the research team. A box for free text comments was additionally included and respondents’ comments were considered by the research team (see Additional file 4: Appendix 4).

A related matter is the composition of participants [29]. Despite being confident that the various rounds allowed the views of a qualified group of experts to be voiced, it could be argued that the participation rate was relatively modest with approximately 15% of the total membership of the respective groups (BASP and BSNR) participating in the final ranking exercise. Furthermore, as eleven BASP members participated in the two initial Delphi rounds, and some of the same respondents could participate in the final ranking exercise by the wider BASP membership. However, in a Delphi and ranking exercise the term “representativeness” is not reflective of how this term is understood within conventional survey techniques. Complex treatment techniques in health care such as thrombectomy are surrounded by uncertainty and can only be addressed by a group of clinicians with sufficient experience and knowledge in this field [30]. Although acknowledging that the study may not be representative of the full spectrum of stroke-related expertise and perspectives in England, it is notable that participants had both extensive experience of treating LAOS patients on a day-to-day basis – including many with more than 10 years of clinical experience – but were also geographically dispersed – with respondents’ from all different regions of England.

The study team also approached the Royal College of Emergency Medicine (RCEM) regarding their potential involvement in the final ranking exercise. However, in discussion with the College we were unable to identify a process that would permit the questionnaire to be sent to the small minority of emergency department physicians with direct expertise/routine involvement in management of LAOS patients (and who make IV thrombolysis decisions – an even smaller minority). A previous position statement from the RCEM has recognised that although emergency medicine doctors are well suited to perform complex treatments such as IV thrombolysis in certain circumstances, this is not considered part of their “core” work responsibilities [31].

Existing workload pressures may have affected the options chosen by participants. It is illustrative that selective transfer to nearest neuroscience centre for “expert thrombectomy” received a 100% approval rate in the first two Delphi rounds with BASP Clinical Standards and/or Executive members from NHS stroke clinical services that deliver 24/7 IV thrombolysis, yet changed dramatically to a significant proportion either disapproving or being neutral to the statement in the ranking exercise with wider BASP membership and the BSNR members. It could be postulated that this change was due to the two Delphi rounds consisting of senior physicians who are comfortable with a clinical selection process rather than the additional novel imaging workload that is likely to fall on them with the other options. Wider representation of BASP and BSNR members in the final ranking exercises with the inclusion of neuroradiologists who, it could be postulated, would predictably rate clinical selection option lower than options that are based largely on brain imaging.

Additionally, asking any healthcare professional about the future organisation of services may be somewhat restricted by current service configurations which, to a greater or lesser extent, influence their thinking when ranking options. Despite their experience in the field putting the consulted experts in a prime position to assess what has worked or not in the past and at present, there are limits to peoples’ abilities or willingness to comprehend configurations and practices beyond those to which they have become accustomed. For instance, the advanced imaging options in the final ranking exercises may have been deemed less favourable due to the current issues facing many NHS trusts in different parts of the country in obtaining advanced imaging of an adequate standard and on time [17]. Realities of the present situation potentially inhibit innovative and more effective solutions from being considered as future scenarios. Consequently, participants might assign a low ranking to certain options and/or will be deterred from proposing constructive changes. It may be imperative to invest in other and supplementary forms of evidence (see ‘further research’ below) to allow for the consideration of more radical changes to current services than are likely to be suggested through consulting healthcare professionals in a Delphi and ranking exercise alone.

### Clinical implications of findings

Enablers to implementation of the simple imaging driven IAT pathway are driven by a perception that a clinical and simple imaging driving pathway is a pragmatic approach, with evidence of feasibility within the NHS for improving outcomes without compromising safety [11]. This option may also be optimally acceptable as it was considered to expedite patient transfer and would not impact negatively on the relaying of CT/CTA images to neuroscience centres and concomitant door-to-treatment times.

Emerging evidence from trials such as the DAWN-trial showing effectiveness of thrombectomy beyond the six-to-eight-hour window and up to 24 h after stroke onset (relating to functional outcomes at 90 days) [32], has implications for the clinical significance of the finding from this Delphi and ranking exercise. However, the findings from the DAWN-trial do not represent the end of the much-cited tenet in stroke care that “time is brain”. Against the backdrop of the emergence of new evidence from the DAWN-trial, the potential for more favourable outcomes of earlier thrombectomy, and the persisting importance of time to treatment in stroke care, should still be considered [33, 34]. For this Delphi and ranking exercise, nevertheless, we cannot rule out that awareness of the emerging DAWN-trial findings amongst respondents (which had not been revealed at the time of conducting the present study) may have impacted on ratings assigned to the different propositions.

A potential barrier to implementing the simple imaging driven IAT pathway is that sites would need to be able to both undertake rapid CT/CTA and provide an expeditious transfer of patients to thrombectomy centres. Without providing efficient links between these components, the clinical effectiveness of thrombectomy services as demonstrated in available evidence [1–15] may not be replicated in routine clinical practice. Implementation barriers to achieving this is the documented variability of stroke care provision across England – both at the pre-hospital and hospital level – relating to costs, funding constraints, geographical location, availability of scanning facilities, achieving outside “office hours” coverage and skill mix of local clinical teams. For example, a review of organisational models of acute stroke care identified regional variation in capacity, expertise and a fragmented approach to organisation of stroke care as barriers to implementation of improved thrombolysis provision [35]. These barriers have important implications for the feasibility of a simple imaging driven IAT pathway in different localities, such as known time delays between hospital transfers [17, 36]. Scope for regional/local flexibility needs to be built into the design and implementation of the preferred option in regards to the assessment of patients/imaging examinations from individual trusts/centres, and the support of rapid and accurate imaging in order to transfer all LAOS patients appropriately.

Staff training in interpretation and decision making about thrombectomy should also be a priority. This would include training for Emergency Medical Services in the identification/presentation of patients eligible for endovascular treatment [23] and available services for inter-professional training for all staff, as well as continued professional development in the light of technological innovations and establishment of additional IT infrastructure. Concurrently, it is important that any staff training does not impact upon the usual services that either trainers or trainees would typically deliver to patients [36].

### Further research

Amidst funding constraints and the need to prioritise resources, an extensive evidence base provides a foundation upon which decision makers can formulate more persuasive arguments for the reconfiguration of services. One component of the evidence base is the implementation preferences and considerations of those who deliver the services in their routine day-to-day clinical practices. Whilst arguing for the value of Delphi methods in providing this key information, we are not advocating for an *exclusive* reliance on the perspectives of the particular subset of stroke experts represented in this study alone. Further research is needed to evaluate the potential barriers/implications of the simple imaging driven IAT pathway as outlined above and to find practical solutions of addressing these. To further inform policymaking, for instance, the unique insights into the perspectives of stroke experts obtained from this study can be contextualised alongside other sources of evidence such as RCTs that assess clinical outcomes of thrombectomy by comparing organisation and transfer methods of services [37]. In addition, in the case of LAOS more research is needed to assess the clinical and cost-effectiveness of the preferred option identified in our study compared to the other options that attracted high approval rankings, but did not reach consensus. There has been a relative paucity of research on the prevalence of the implementation alternatives associated with their costs [38, 39]; however the results of this Delphi and ranking exercise (and a parallel exercise to elicit preferences of stroke survivors/their carers and the public on service configurations for delivery of thrombectomy) will be used to inform/parameterise a health economic model to evaluate the cost-utility (estimated using a quality adjusted life years framework) of the simple imaging driven IAT pathway and alternative service configurations in England. This will provide policymakers and commissioners with data on the flexibility of service specifications that take into account variations in distance and travel time to appropriate centres, as well as patient characteristics and skill mix of clinical teams and infrastructure/resources needed to provide a comprehensive thrombectomy service.

## Conclusions

This Delphi and ranking exercise with clinical experts on the future delivery of thrombectomy services in England has established consensus on a “simple” imaging driven option, which advocates the secondary transfer of patients with LAOS for thrombectomy based on local CT/CTA. IT facilities and adequate staffing level/expertise are needed to enable rapid review by (neuro) interventionists and, where appropriate, secondary transfer of LAOS patients to thrombectomy capable centres. The methods employed here offer the potential to provide supplementary information to aid policymakers and practitioners making decisions on service reconfiguration, including the provision of data on factors that will impact on implementation for the prospective reconfiguration of stroke services. These data can be assessed alongside other sources of evidence and may, in particular, complement trials on the effectiveness of thrombectomy.

## Additional files

Additional file 1: **Appendix 1.** Format of Ranking Exercise with wider British Association of Stroke Physicians (BASP) members. (DOCX 22 kb)

Additional file 2: **Appendix 2.** Format of Ranking Exercise with British Society of Neuroradiologists (BSNR). (DOCX 21 kb)

Additional file 3: **Appendix 3.** Additional Summary Statistics for Panellist Responses to each option. (DOCX 25 kb)

Additional file 4: **Appendix 4.** Conceptual Content Analysis of Free Text Comments from Ranking Exercise. (DOCX 16 kb)

Additional file 5: **Appendix 5.** Profile of Participants - British Association of Stroke Physicians (BASP) Round 1. (DOCX 15 kb)

## Abbreviations

ASPECTS: Alberta Stroke Program Early CT Score
BASP: British Association of Stroke Physicians
BSNR: British Society of Neuroradiologists
CT: Computed Tomography
IAT: Intra-Arterial mechanical Thrombectomy
INR: Interventional NeuroRadiology
IV: IntraVenous
LAOS: Large Artery Occlusive Stroke
NHS: National Health Service
RCEM: Royal College of Emergency Medicine
RCTs: Randomised Controlled Trials
