# Supplementary material for: A Delphi study and ranking exercise to support commissioning services: future delivery of Thrombectomy services in England
Source: BMC Health Serv Res. 2018 Feb 22;18:135. doi: 10.1186/s12913-018-2922-3 (PMC5824465; doi:10.1186/s12913-018-2922-3)
Supplement: Supplementary file 1 — Appendix 1. Format of Ranking Exercise with wider British Association of Stroke Physicians (BASP) members. (DOCX 22 kb) [file 12913_2018_2922_MOESM1_ESM.docx]

**Supplementary Material Appendix 1.**

**Ranking Exercise with wider British Association of Stroke Physicians (BASP) members**

**Thrombectomy Provision- Rankings Exercise**

Thank you for agreeing to participate in this study. We would be grateful if you could please complete the following baseline questions:

**1. Years as a stroke physician:**

[ ] 0-5 years

[ ] 5-10 years

[ ] 10+ years

**2. Region where you currently work**:

[ ] North East England

[ ] North West England

[ ] Yorkshire and the Humber

[ ] East Midlands, England

[ ] West Midlands, England

[ ] East of England

[ ] London, England

[ ] South East England

[ ] South West England

**3. Do you currently have arrangements in place to refer patients for thrombectomy?**

Yes [ ]

No [ ]

If Yes, are these

Formal? [ ]

or

ad hoc? [ ]

**Thank you – please go to the next page for the ranking exercise**

**Ranking Exercise**

Below we present a description of remaining potential options for triaging patients for thrombectomy. Please score each of the options using a 7-point Likert scale:

| **1** | **2** | **3** | **4** | **5** | **6** | **7** |
| --- | --- | --- | --- | --- | --- | --- |
| **very strongly disapprove** | **quite strongly disapprove** | **disapprove** | **neutral** | **approve** | **quite strongly approve** | **very strongly approve** |

Using your experience and judgement, please take the following elements into consideration when assigning scores to the 3 options:

1. **Availability**
2. **Practicality/Deliverability**
3. **Cost** (including of any additional software or hardware likely to be required in your region)

**OPTION 1: “SIMPLE” IMAGING DRIVEN (Time critical pathway)**

**Patients with large artery occlusive stroke are transferred to nearest [neuroscience] centre for thrombectomy based on local CT/CTA alone**.

- *37% of all stroke patients arrive at hospital within 4h with ischaemic stroke of known onset time. ~40-50% of patients have large artery occlusive strokes.*
- *Adjunctive IAT approach is proven (level 1 evidence) to increase mRS 0-2 by 12% to 14% with benefit across the Rankin scale of shift to reduced disability*
- Facilities will need to be available for the neurointerventionist to rapidly review CT/CTA prior to accepting a referral. This may require additional IT infrastructure
- Responsibility for formal reporting will be with the centre acquiring the CT/CTA images unless other contractual arrangements are formally agreed.

| **1** | **2** | **3** | **4** | **5** | **6** | **7** |
| --- | --- | --- | --- | --- | --- | --- |
| **very strongly disapprove** | **quite strongly disapprove** | **disapprove** | **neutral** | **approve** | **quite strongly approve** | **very strongly approve** |

**OPTION 2: ADVANCED IMAGING DRIVEN**

**Patients are transferred to nearest [neuroscience] centre for thrombectomy based on advanced imaging obtained at referring hospital**

- - *Selective brain tissue viability assessment approach to IAT is proven (level 1 evidence) to increase mRS 0-2 by 24% to 31% with benefit across the Rankin scale of shift to reduced disability*
  - *All RCT results are based on expert interpretation of advanced imaging as triage for intra-arterial thrombectomy*
  - *Facilities will need to be available for the neurointerventionist to rapidly review imaging prior to accepting a referral. This will require additional IT infrastructure*
  - *Responsibility for formal reporting will be with the centre acquiring the imaging unless other contractual arrangements are formally agreed.*

| **1** | **2** | **3** | **4** | **5** | **6** | **7** |
| --- | --- | --- | --- | --- | --- | --- |
| **very strongly disapprove** | **quite strongly disapprove** | **disapprove** | **neutral** | **approve** | **quite strongly approve** | **very strongly approve** |

**OPTION 3: CLINICAL JUDGEMENT DRIVEN (Time critical pathway)**

**Selective transfer to nearest on call [neuroscience] thrombectomy centre for expert thrombectomy**

- **This is a flexible clinical judgement driven referral route** – so that for example if plain CT shows an obvious hyper-dense MCA sign, the ASPECTS score is good (7+) & NIHSS is ≥6, referral for thrombectomy is made without CTA if obtaining such locally would add significant delays. However, this may add delay downstream in the pathway for thrombectomy as a second CT scanner visit will be required on arrival at receiving hospital
- *This may entail networking of Neurorinterventional units to deliver 24/7 cover sooner- with some longer transfer times, but does mean the efficacy data from RCTs can be applied (underpinned by data for UK centres from the PISTE trial)*

| **1** | **2** | **3** | **4** | **5** | **6** | **7** |
| --- | --- | --- | --- | --- | --- | --- |
| **very strongly disapprove** | **quite strongly disapprove** | **disapprove** | **neutral** | **approve** | **quite strongly approve** | **very strongly approve** |

**Please use the text box below for any comments you may have about the 3 options for delivering thrombectomy:**
