# Supplementary material for: A Delphi study and ranking exercise to support commissioning services: future delivery of Thrombectomy services in England
Source: BMC Health Serv Res. 2018 Feb 22;18:135. doi: 10.1186/s12913-018-2922-3 (PMC5824465; doi:10.1186/s12913-018-2922-3)
Supplement: Supplementary file 2 — Appendix 2. Format of Ranking Exercise with British Society of Neuroradiologists (BSNR). (DOCX 21 kb) [file 12913_2018_2922_MOESM2_ESM.docx]

**Supplementary Material Appendix 2.**

**Ranking Exercise with British Society of Neuroradiologists (BSNR)**

**Thrombectomy Provision- Rankings Exercise**

Thank you for agreeing to participate in this study.

We would be grateful if you could please complete the following baseline questions:

**1. Years as a consultant neuroradiologist:**

[ ] 0-5 years

[ ] 5-10 years

[ ] 10+ years

**2. Region where you currently work**:

[ ] North East England or Yorkshire and the Humber

[ ] North West England

[ ] East or West Midlands

[ ] East of England or South East England

[ ] London

[ ] South West England

**3. Do you currently undertake regular cerebral vascular interventions?**

YES [ ]

NO [ ]

**Thank you – please go to the next page for the ranking exercise**

**Ranking Exercise**

Below we present a description of 4 potential options for triaging patients for thrombectomy. Please score each of the options using a 7-point Likert scale:

| **1** | **2** | **3** | **4** | **5** | **6** | **7** |
| --- | --- | --- | --- | --- | --- | --- |
| **very strongly disapprove** | **quite strongly disapprove** | **disapprove** | **neutral** | **approve** | **quite strongly approve** | **very strongly approve** |

We want you, using your experience & judgement to take the following elements into consideration when assigning scores to the options:

- Availability
- Practicality/Deliverability
- Cost (including of any additional software or hardware likely to be required in your region)

Whilst options 2 & 3 are both “Advanced Imaging Triage” they may differ in deliverability, cost & practicality so they have been separated out for this exercise

There is of course uncertainty over the strength of evidence supporting either option

**OPTION 1**

**Patients are transferred for thrombectomy based on local CT/CTA alone**.

- Facilities will need to be available for the neurointerventionist to rapidly review these prior to accepting a referral. This may require additional IT infrastructure
- Responsibility for formal reporting will be with the centre acquiring the CT/CTA images unless other contractual arrangements are formally agreed.

| **1** | **2** | **3** | **4** | **5** | **6** | **7** |
| --- | --- | --- | --- | --- | --- | --- |
| **very strongly disapprove** | **quite strongly disapprove** | **disapprove** | **neutral** | **approve** | **quite strongly approve** | **very strongly approve** |

**OPTION 2**

**Patients are transferred for thrombectomy based on formal ASPECTS & Collateral Scoring in addition to confirming large artery occlusion present - “Advanced Imaging Triage ACS”**

- This reflects evidence of ESCAPE trial. Footnotes above also apply to all these options

| **1** | **2** | **3** | **4** | **5** | **6** | **7** |
| --- | --- | --- | --- | --- | --- | --- |
| **very strongly disapprove** | **quite strongly disapprove** | **disapprove** | **neutral** | **approve** | **quite strongly approve** | **very strongly approve** |

**OPTION 3**

**Patients are transferred for thrombectomy based on CT Perfusion parameters in addition to confirming large artery occlusion present - “Advanced Imaging Triage PERFUSION”**

- This reflects evidence of EXTEND/SWIFT PRIME trials. Footnotes to option 1 also apply
- This may require a region wide adoption of a standardised protocol & software such as RAPID or OLEA

| **1** | **2** | **3** | **4** | **5** | **6** | **7** |
| --- | --- | --- | --- | --- | --- | --- |
| **very strongly disapprove** | **quite strongly disapprove** | **disapprove** | **neutral** | **approve** | **quite strongly approve** | **very strongly approve** |

**OPTION 4**

**Selective transfer to nearest on call neuroscience centre for “expert thrombectomy**

- **This is a flexible clinical judgement driven referral route** – so that for example if plain CT shows an obvious hyper-dense MCA sign, the ASPECTS score is good (7+) & NIHSS is ≥6, referral for thrombectomy is made without CTA, which may add delay to the pathway to thrombectomy

| **1** | **2** | **3** | **4** | **5** | **6** | **7** |
| --- | --- | --- | --- | --- | --- | --- |
| **very strongly disapprove** | **quite strongly disapprove** | **disapprove** | **neutral** | **approve** | **quite strongly approve** | **very strongly approve** |

**Please use the text box below for any comments you may have about the potential options for delivering thrombectomy:**
