# Supplementary material for: A Delphi study and ranking exercise to support commissioning services: future delivery of Thrombectomy services in England
Source: BMC Health Serv Res. 2018 Feb 22;18:135. doi: 10.1186/s12913-018-2922-3 (PMC5824465; doi:10.1186/s12913-018-2922-3)
Supplement: Supplementary file 3 — Appendix 3. Additional Summary Statistics for Panellist Responses to each option. (DOCX 25 kb) [file 12913_2018_2922_MOESM3_ESM.docx]

**Additional file 3.**

**Additional Summary Statistics for Panellist Responses to each option**

| **1** | **2** | **3** | **4** | **5** | **6** | **7** |
| --- | --- | --- | --- | --- | --- | --- |
| **very strongly disapprove** | **quite strongly disapprove** | **disapprove** | **neutral** | **approve** | **quite strongly approve** | **very strongly approve** |

**Second Delphi round with British Association of Stroke Physicians (BASP) Clinical Standards and/or Executive Membership (n=11)**

|  | **Likert Scale Responses** | | | | | | |
| --- | --- | --- | --- | --- | --- | --- | --- |
| **Proposition** | **Median** | **IQR** | **LQ** | **UQ** | **Mode(s)** | **Min** | **Max** |
| 2. Any local provider delivers IAT on a formal rota | 2 | 3 | 2 | 5 | 2 | 2 | 5 |
| 4. Transfer to nearest primary coronary percutaneous intervention unit and shared care with stroke physicians | 3 | 2 | 2 | 4 | 3 | 1 | 7 |
| 5. Ambulance bypass for all acute stroke patients of known time onset to comprehensive stroke unit where advanced imaging and “expert intra-arterial thrombectomy [IAT]” are available 24/7 | 3 | 1 | 3 | 4 | 3 | 2 | 5 |
| 6. Local CT and transfer all patients with NIHSS ≥ 10 to the nearest neuroscience centre for interventional neuroradiologist delivered “expert thrombectomy” ** | 5 | 2 | 4 | 6 | 4,5,6 | 3 | 6 |
| **7. Local CT/CTA then transfer all large artery occlusive stroke patients to nearest neuroscience centre for interventional neuroradiologist delivered “expert thrombectomy” **** | **6** | **2** | **5** | **7** | **6** | **5** | **7** |
| 8. Local advanced imaging then selective transfer to nearest neuroscience centre for “expert thrombectomy” | 5 | 2 | 4 | 6 | 6 | 3 | 7 |
| **9. Local CT/CTA then transfer large artery occlusive stroke patients to nearest neuroscience centre for advanced imaging and “expert thrombectomy”** | **6** | **1** | **5** | **6** | **6** | **4** | **7** |
| 10. Advanced imaging performed locally but interpreted centrally by Neuroradiology then selective transfer to nearest neuroscience centre for “expert thrombectomy” | 6 | 2 | 4 | 6 | 6 | 3 | 7 |
| **11. Selective transfer to nearest on call neuroscience centre for “expert thrombectomy”** | **6** | **1** | **5** | **6** | **6** | **5** | **7** |

****** N=10

IQR = interquartile range

LQ = lower quartile

UQ = upper quartile

NB: Propositions that achieved consensus approval have been highlighted in bold text.

**Ranking Exercise with wider BASP membership (n=43)**

|  | **Likert Scale Responses** | | | | | | |
| --- | --- | --- | --- | --- | --- | --- | --- |
| **Proposition** | **Median** | **IQR** | **LQ** | **UQ** | **Mode(s)** | **Min** | **Max** |
| 1. **Patients with large artery occlusive stroke are transferred to nearest [neuroscience] centre for thrombectomy based on local CT/CTA alone** | **6** | **0** | **6** | **6** | **6** | **2** | **7** |
| 1. Patients are transferred to nearest [neuroscience] centre for thrombectomy based on advanced imaging obtained at referring hospital | 4 | 2 | 3 | 5 | 4 | 1 | 7 |
| 1. Selective transfer to nearest on call [neuroscience] thrombectomy centre for expert thrombectomy | 4 | 3 | 2 | 5 | 2 and 4 | 1 | 7 |

IQR = interquartile range

LQ = lower quartile

UQ = upper quartile

NB: Propositions that achieved consensus approval have been highlighted in bold text.

**Ranking Exercise with the British Society of Neuroradiologists (BSNR) (n=21)**

|  | **Likert Scale Responses** | | | | | | |
| --- | --- | --- | --- | --- | --- | --- | --- |
| **Proposition** | **Median** | **IQR** | **LQ** | **UQ** | **Mode(s)** | **Min** | **Max** |
| 1. **Patients are transferred for thrombectomy based on local CT/CTA alone** | **6** | **2** | **5** | **7** | **7** | **1** | **7** |
| 1. Patients are transferred for thrombectomy based on formal ASPECTS & Collateral Scoring in addition to confirming large artery occlusion present - “Advanced Imaging Triage ACS”****** | 4 | 2.75 | 3.25 | 6 | 4 | 3 | 7 |
| 1. Patients are transferred for thrombectomy based on CT Perfusion parameters in addition to confirming large artery occlusion present - “Advanced Imaging Triage PERFUSION”****** | 3 | 1.75 | 2 | 3.75 | 3 | 1 | 6 |
| 1. Selective transfer to nearest on call neuroscience centre for “expert thrombectomy” | 5 | 4 | 3 | 7 | 3,5,7 | 2 | 7 |

****** N=20

IQR = interquartile range

LQ = lower quartile

UQ = upper quartile

NB: Propositions that achieved consensus approval have been highlighted in bold text.
