# Supplementary material for: A Delphi study and ranking exercise to support commissioning services: future delivery of Thrombectomy services in England
Source: BMC Health Serv Res. 2018 Feb 22;18:135. doi: 10.1186/s12913-018-2922-3 (PMC5824465; doi:10.1186/s12913-018-2922-3)
Supplement: Supplementary file 4 — Appendix 4. Conceptual Content Analysis of Free Text Comments from Ranking Exercise. (DOCX 16 kb) [file 12913_2018_2922_MOESM4_ESM.docx]

**Additional file 4.**

**Conceptual Content Analysis of Free Text Comments from Ranking Exercise**

Themes within the free text comments associated with the simple imaging driven Intra-Arterial mechanical Thrombectomy (IAT) pathway (*patients with large artery occlusive stroke are transferred to nearest [neuroscience] centre for thrombectomy based on local CT/CT* *Angiography alone)* identified a range of views on the benefits/strengths and potential drawbacks/issues to be addressed that were associated with this option.

**Benefits/strengths**

A key theme was that facilities and adequate staffing level/expertise need to be available to rapidly review CT/CT Angiography prior to accepting a referral. Respondents generally indicated a preference for a simple imaging driven IAT pathway based on the assertion that it is the simplest and most pragmatic approach that appears to be working well already in the majority of National Health Service (NHS) trusts in England. One respondent expressed disappointment that this option “is not universally available yet” but “hopefully will be soon” (British Society of Neuroradiologists (BSNR) member with 10+ years of experience).

A simple imaging driven IAT pathway was not only underscored as the most cost-effective – benefitting from currently available infrastructure and routine practices – but also as the most clinically effective option. As the following British Association of Stroke Physicians (BASP) member with more than 10 years of experience as a stroke physician expressed it; “Option 1 is the most viable option both clinically and cost”. A simple imaging driven IAT pathway was considered to provide “excellent clinical outcomes for selected patients” in another of the respondents’ trusts (BASP member with 0-5 years of experience). In situations where local stroke expertise was limited, the perceived simplicity of implementation also attracted views that a simple imaging driven IAT pathway was the safest option. The following respondent emphasised how this pathway “places the gate keeping onus on the receiver but may work better (and be safer) in situations where local stroke expertise are limited” (BASP member with 5-10 years of experience, brackets in original).

Moreover, respondents frequently stated their preference for a simple imaging driven IAT pathway due to critical time considerations in the management and treatment of stroke. One respondent described it as the “best course balancing need for speed with definite evidence of large artery occlusion” (BASP member with 10+ years of experience). Similarly, another BASP member with 10+ years of experience as a stroke physician favoured this option as it facilitated “agreed rapid repatriation even on the same day”, while a third BASP member with the same number of years of experience asserted that “sending images across to a stroke centre doesn’t usually take very long”.

**Potential drawbacks/issues that need to be addressed**

Several respondents expressed concerns about resources to implement a simple imaging driven IAT pathway; in particular with reference to universal out-of-hours CT Angiography not being available in all trusts. For example, one respondent stated that out-of-hours availability was restricted to general radiology and physician cover with the support from telemedicine (BASP member with 10+ years of experience). Further implementation challenges related to the need for IT infrastructure to be updated and made more readily available. Indeed, one respondent reported considerable heterogeneity between scanners (BASP member with 5-10 years of experience).

Heterogeneity between trusts was also identified, with smaller and/or rural trusts often being less resource-intensive and additionally constrained by significant travel time to the nearest neuroscience centre. For example, a respondent working in a rural hospital reported that some patients had to travel more than an hour by ambulance to reach the nearest neuroscience centre (BASP member with 10+ years of experience).

Several respondents emphasised that interpreting CT/CT Angiography images is dependent upon the skill mix of the clinical team. Conversely, one respondent suggested that CT Angiography “can be done at the same time as the plain CT and does not add too much time and could easily be reported by the local team” (BASP member with 10+ years of experience); however several respondents generally considered CT Angiography to be a more advanced technique. As such, the issue of which personnel should be authorised to make decisions about transfer for thrombectomy would appear to be contentious. The need for transfer to be based on agreed pathways and/or by consultants only was noted, whereas it was also suggested that less experienced staff could make such decisions with adequate support/training to interpret CT Angiography images (e.g. as facilitated in-house or as provided by collaborating neuroscience centres). One respondent suggested that previous training in reading plain CT imaging for intravenous (IV) thrombolysis would be sufficient to provide transferable skills for interpretation of CT Angiography images (BASP member with 10+ years of experience). A further respondent asserted that by allowing clinical input, the simple imaging driven IAT pathway nullifies the need for additional multimodal CT Angiography scans to be performed (BASP member with 5-10 years of experience).
