# Supplementary material for: A Delphi study and ranking exercise to support commissioning services: future delivery of Thrombectomy services in England
Source: BMC Health Serv Res. 2018 Feb 22;18:135. doi: 10.1186/s12913-018-2922-3 (PMC5824465; doi:10.1186/s12913-018-2922-3)
Supplement: Supplementary file 5 — Appendix 5. Profile of Participants - British Association of Stroke Physicians (BASP) Round 1. (DOCX 15 kb) [file 12913_2018_2922_MOESM5_ESM.docx]

**Additional file 5.**

**Profile of Participants - British Association of Stroke Physicians (BASP) Round 1 (N=15)**

|  | | **Frequency** | **Percent** |
| --- | --- | --- | --- |
| **Years as a stroke consultant** | 0 to 5 years | 1 | 7 |
|  | 5 to 10 years | 4 | 27 |
|  | 10+ years | 10 | 67 |
| **Region where you currently work** | North East England | 2 | 13 |
|  | Yorkshire and the Humber | 2 | 13 |
|  | East Midlands, England | 1 | 7 |
|  | West Midlands, England | 1 | 7 |
|  | East of England | 1 | 7 |
|  | London, England | 4 | 27 |
|  | South East England | 2 | 13 |
|  | South West England | 1 | 7 |
|  | Missing data | 1 | 7 |

NB: percentages may not equal 100 due to rounding.
